# Astaxanthin intake attenuates muscle atrophy caused by immobilization in rats

**DOI:** 10.14814/phy2.12885

**Published:** 2016-08-01

**Authors:** Tsubasa Shibaguchi, Yusuke Yamaguchi, Nobuyuki Miyaji, Toshinori Yoshihara, Hisashi Naito, Katsumasa Goto, Daijiro Ohmori, Toshitada Yoshioka, Takao Sugiura

**Affiliations:** ^1^ Organization of Frontier Science and InnovationKanazawa UniversityKanazawa CityIshikawaJapan; ^2^ Department of Exercise and Health SciencesFaculty of EducationYamaguchi UniversityYamaguchi CityYamaguchiJapan; ^3^ Toyo Koso Kagaku Co. Ltd.Urayasu CityChibaJapan; ^4^ Graduate School of Health and Sports ScienceJuntendo UniversityInzai CityChibaJapan; ^5^ Graduate School of Health SciencesToyohashi SOZO UniversityToyohashi CityAichiJapan; ^6^ Department of ChemistrySchool of MedicineJuntendo UniversityInzai CityChibaJapan; ^7^ Hirosaki Gakuin UniversityHirosaki CityAomoriJapan

**Keywords:** Astaxanthin, muscle atrophy, oxidative stress, protease

## Abstract

Astaxanthin is a carotenoid pigment and has been shown to be an effective inhibitor of oxidative damage. We tested the hypothesis that astaxanthin intake would attenuate immobilization‐induced muscle atrophy in rats. Male Wistar rats (14‐week old) were fed for 24 days with either astaxanthin or placebo diet. After 14 days of each experimental diet intake, the hindlimb muscles of one leg were immobilized in plantar flexion position using a plaster cast. Following 10 days of immobilization, both the atrophic and the contralateral plantaris muscles were removed and analyzed to determine the level of muscle atrophy along with measurement of the protein levels of CuZn‐superoxide dismutase (CuZn‐SOD) and selected proteases. Compared with placebo diet animals, the degree of muscle atrophy in response to immobilization was significantly reduced in astaxanthin diet animals. Further, astaxanthin supplementation significantly prevented the immobilization‐induced increase in the expression of CuZn‐SOD, cathepsin L, calpain, and ubiquitin in the atrophied muscle. These results support the postulate that dietary astaxanthin intake attenuates the rate of disuse muscle atrophy by inhibiting oxidative stress and proteolysis via three major proteolytic pathways.

## Introduction

Skeletal muscles produce abundant reactive oxygen species (ROS) during both aerobic contractions and inactivity, such as bed rest and limb immobilization (Powers et al. 2005, 2007, 2011; Powers and Jackson 2008). Indeed, Kondo et al. provided the first evidence regarding the relationship between oxidative stress and disuse muscle atrophy (Kondo et al. 1991), and then their group also indicated that generation of various types of ROS is promoted in atrophied muscle induced by immobilization (Kondo et al. 1992, 1993a,b, 1994). In addition, oxidative stress has been shown to participate in the activation of proteolysis via three major proteolytic pathways, lysosomal proteases (e.g., cathepsin L), Ca^2+^‐activated proteases (i.e., calpain), and the ubiquitin–proteasome system, all of which are activated during disuse muscle atrophy (Powers et al. 2005, 2007). Thus, it is considered that disuse‐induced oxidative stress in skeletal muscle contributes to muscular atrophy via the activation of these proteolytic pathways.

A number of studies have demonstrated that supplementation of various types of antioxidants is one of the effective countermeasures against disuse muscle atrophy with increasing oxidative stress. For example, vitamin E administration attenuates oxidative stress and muscle atrophy induced by immobilization (Kondo et al. 1991, 1992). Servais et al. (2007) also reported inhibitory effects of vitamin E on muscular atrophy and up‐regulation of the concentration of thiobarbituric acid‐reactive substances (TBARS), an indicator of lipid peroxidation, and the mRNA expression of calpains and E3 ubiquitin ligases in response to hindlimb unloading. In addition, Ikemoto et al. (2002a) showed that high‐dose supplementation of cysteine (140 mg per rat) inhibited hindlimb unloading‐induced muscle atrophy thorough a suppression of oxidative stress and protein ubiquitination. Furthermore, we previously reported that estrogen administration to male rats attenuated the rate of muscle atrophy induced by immobilization due, in part, to reductions of the immobilization‐induced increase of CuZn‐superoxide dismutase (CuZn‐SOD), a cytosolic antioxidant enzyme which catalyses the dismutation of superoxide anions, and calpain levels (Sugiura et al. 2006). It is noteworthy, however, that whereas administration of these antioxidants can attenuate disuse muscle atrophy, some researchers revealed that antioxidant treatments had no effect on reducing it (Ikemoto et al. 2002b; Koesterer et al. 2002). Hence, further explanation is required to clarify what kinds of antioxidants are highly effective for preventing disuse muscle atrophy.

Astaxanthin, a red carotenoid pigment, is a biological antioxidant that exists naturally in algae, fish, crustacean, and birds (Guerin et al. 2003). Astaxanthin has been shown to have powerful antioxidant activities by scavenging and quenching ROS and free radicals (superoxide anion, hydrogen peroxide, singlet oxygen, etc.) and inhibiting lipid peroxidation in vitro (Kurashige et al. 1990; Miki 1991; Kobayashi 2000). In particular, the inhibitory effect of astaxanthin against the action of free radicals and the lipid peroxidation is approximately more than 100 times greater than that of vitamin E (Kurashige et al. 1990; Miki 1991). These highly antioxidant activities of astaxanthin may be attributed to the unique structure of the terminal ring moiety, which is able to trap radicals both at the surface and in the inside of the phospholipid membrane (Goto et al. 2001). In reference to astaxanthin and skeletal muscle, Aoi et al. (2003) provided the first evidence that astaxanthin accumulated in mouse skeletal muscle after 3 weeks of dietary intake of astaxanthin, and this treatment attenuated oxidative damage to lipids and DNA, as well as expression of inflammatory mediators, in the muscle tissue after intense exercise. We and others also showed that astaxanthin supplementation reduced up‐regulation of CuZn‐SOD expression and/or accumulation of ROS in response to hindlimb unloading with or without attenuating soleus muscle atrophy (Kanazashi et al. 2013, 2014; Yoshihara et al. 2016). These findings suggest that dietary astaxanthin intake may be an effective countermeasure against muscle damage and disuse‐induced muscle atrophy.

However, as described above, the attenuating effect of astaxanthin intake on disuse muscle atrophy remains debatable. In addition, there is no evidence regarding the influences of astaxanthin supplementation on muscle mass and proteolytic pathways during immobilization‐induced disuse muscle atrophy. Therefore, this study was performed to investigate the effects of dietary astaxanthin intake on disuse muscle atrophy induced by immobilization of rat hindlimb from the standpoint of oxidative stress and three major proteolytic pathways.

## Materials and Methods

### Animals and experimental design

All experimental procedures followed the *Guide for the Care and Use of Laboratory Animals* of the Physiological Society of Japan and were approved by the Committee on Animal Care and Use in Yamaguchi University. Male Wistar strain rats (*n* = 23, 14 weeks of age) were used in this study. After 1 week of acclimation, rats were randomly assigned into three groups: placebo diet (Placebo, *n* = 7), 0.04% astaxanthin diet (Ax 0.04%, *n* = 8), and 0.2% astaxanthin diet (Ax 0.2%, *n* = 8). Throughout the experiments, the rats were individually housed in a climate‐controlled room (26 ± 1°C, 56 ± 1% relative humidity and 12:12‐h light–dark photoperiod) and given their respective diets and water ad libitum.

Astaxanthin (BioAstin) and placebo powders were obtained from Toyo Koso Kagaku Co. Ltd., Japan. The astaxanthin included in the powder is derived from *Haematococcus pluvialis*, which is the richest source of natural astaxanthin (Guerin et al. 2003). Either astaxanthin or placebo powder was mixed with a CE‐2 powder diet (CLEA Japan). The composition of each experimental diet is shown in Table 1. In reference to the appropriate timing of antioxidant supplementation against disuse muscle atrophy, Appell et al. (1997) recommended the antioxidant supplementation prior to and during the early phase of immobilization because oxidative stress plays a role in initiating muscle atrophy. Furthermore, we recently reported that dietary astaxanthin intake 2 week prior to and during hindlimb unloading attenuated soleus muscle atrophy in rats (Yoshihara et al. 2016). In this study, therefore, dietary intake of each experimental food was started 14 days before immobilization. Following 14 days of each experimental diet intake, one hindlimb of all rats was immobilized in plantar flexion with plaster (Castlight, ALCARE Co., Ltd., Japan) for 10 days. Food intake was measured daily and the mean intake before (14 days) and during (10 days) the immobilization period was calculated.

**Table 1:** Percent composition of experimental diets

	Placebo	Ax 0.04%	Ax 0.2%
CE‐2 powder	93.880	93.804	93.510
Bio Astin SCE10	0.000	0.040	0.200
Oil	0.039	0.040	0.044
Ester F160	0.217	0.217	0.218
Quillayanin C‐100	0.587	0.588	0.590
Amycol C	1.016	1.018	1.026
Trehalose	3.885	3.917	4.034
Zein DP	0.376	0.376	0.378

Definition of abbreviations: Placebo, placebo diet group; Ax 0.04%, 0.04% astaxanthin diet group; Ax 0.2%, 0.2% astaxanthin diet group.

At the completion of the experiment, rats were killed by an overdose intraperitoneal injection of sodium pentobarbital (100 mg kg^−1^ body weight). After the body weight measurement, plantaris muscles of both legs (immobilized and control) were quickly removed. In contrast to hindlimb unloading model, it was reported that hindlimb immobilization in rats induced an approximately similar degree of atrophy in slow soleus and fast plantaris muscles (Herbison et al. 1978; Thomason and Booth 1990). However, the information about supplementation of antioxidants and immobilization‐induced fast muscle atrophy is limited. In this study, therefore, we used fast plantaris muscle to evaluate the effect of dietary astaxanthin intake on muscle atrophy induced by immobilization. Removed plantaris muscles were carefully weighted, rapidly frozen in liquid nitrogen, and stored at −80°C until analyses. The degree of plantaris muscle atrophy was calculated by the following formula: Percent weight change from contralateral muscle = (contralateral − immobilized)/contralateral plantaris weight × 100.

### Muscle preparation

A portion of plantaris muscles (40–60 mg) was minced and homogenized in ice‐cold homogenization buffer (10 mmol L^−1^ Tris‐HCl pH 7.6, 10 mmol L^−1^ NaCl, and 0.1 mmol L^−1^ EDTA). The homogenates were centrifuged at 1500 ×* g* for 15 min at 4°C, and the protein concentration of the supernatants was determined using a protein assay kit (Bio‐Rad, Richmond, CA).

### Sodium dodecyl sulfate‐polyacrylamide gel electrophoresis (SDS‐PAGE) and western blotting

Protein extracts were solubilized in sample loading buffer (30% glycerol, 5% 2‐mercaptoethanol, 2.3% SDS, 62.5 mmol L^−1^ Tris‐HCl pH 6.8, and 0.05% bromophenol blue) at a concentration of 2.0 mg mL^−1^ and incubated at 60°C for 10 min. Proteins were then separated by 8, 10, or 14% SDS‐PAGE and transferred onto a polyvinylidene difluoride membrane (Millipore, Bedford, MA) using a mini trans‐blot cell (Bio‐Rad) at a constant voltage of 100 V for 60 min at 4°C in transfer buffer (25 mmol L^−1^ Tris‐HCl pH 8.3, 192 mmol L^−1^ glycine, and 20% methanol) (Towbin et al. 1979). After protein transfer, the membranes were blocked for 1 h at room temperature using a blocking buffer [5% nonfat dry milk in Tween‐Tris‐buffered saline (T‐TBS; 20 mmol L^−1^ Tris‐HCl, 150 mmol L^−1^ NaCl, and 0.05% Tween‐20, pH 7.5)]. Following serial washing with T‐TBS, the membranes were incubated overnight at 4°C with primary antibodies to CuZn‐SOD (1:10,000 in T‐TBS; SOD‐101, Stressgen, Victoria, B.C., Canada), cathepsin L (1:100 in T‐TBS containing 5% bovine serum albumin; C2970, Sigma, St. Louis, MO), calpain (*μ*‐ or m‐calpain: 1:1000 in T‐TBS containing 5% bovine serum albumin; C6361, Sigma), ubiquitin (1:250 in T‐TBS; SPA‐200, Stressgen), and heat‐shock protein 72 (HSP72: 1:10,000 in T‐TBS; SPA‐812, Stressgen). After several washes in T‐TBS, membranes were reacted with either alkaline phosphatase‐conjugated anti‐rabbit IgG (A‐3682, Sigma) or anti‐mouse IgG antibody (A‐4312, Sigma) diluted at 1:30,000 in T‐TBS as appropriate for 1 h at room temperature. The membranes were then washed three times in T‐TBS for 5 min each. The protein bands were visualized using an Alkaline Phosphatase Conjugate Substrate Kit (Bio‐Rad). Quantification of each detected protein band was performed using CS Analyzer 2.0 (ATTO, Tokyo, Japan). The protein content was expressed as the relative ratio (%) to the nonimmobilized muscles of the placebo diet group. Glyceraldehyde‐3‐phosphate dehydrogenase (GAPDH: GTX100118, GeneTex, Irvine, CA) was evaluated to confirm the equal protein loading.

### Statistics

All values are reported as means ± standard error (SE). Statistical significance was determined using a one‐way analysis of variance (ANOVA) or two‐way ANOVA followed by Tukey's post hoc test as appropriate. *P *<* *0.05 was considered statistically significant.

## Results

Body weight and food consumption in all experimental groups are shown in Table 2. No significant differences in the body weight among the three groups were observed after 10 days of immobilization. The food consumption in all experimental groups during the immobilization was significantly lower compared with the value in each group before the immobilization (*P *<* *0.05). However, there were no significant differences in the food consumption among the three groups in each period. In this study, the estimated amount of astaxanthin intake was 10.1 ± 0.3 and 6.5 ± 0.5 mg per day per rat in the Ax 0.04% group and 49.6 ± 1.2 and 32.0 ± 1.2 mg per day per rat in the Ax 0.2% group before and during the immobilization period, respectively.

**Table 2:** Body weight and food consumption

	Placebo	Ax 0.04%	Ax 0.2%
Body weight, g	447.3 ± 7.5	440.3 ± 15.3	433.8 ± 6.9
Food consumption, g per day			
Before	24.2 ± 0.5	25.2 ± 0.7	24.8 ± 0.6
During	17.3 ± 1.4*	16.2 ± 1.2*	16.0 ± 0.6*

Values are means ± SE. *n* = 7–8 per group. Before, before hindlimb immobilization period; During, during hindlimb immobilization period. See Table 1 for the other abbreviations. *Significantly different from Before in each group (*P *<* *0.05).

**Figure 1:**
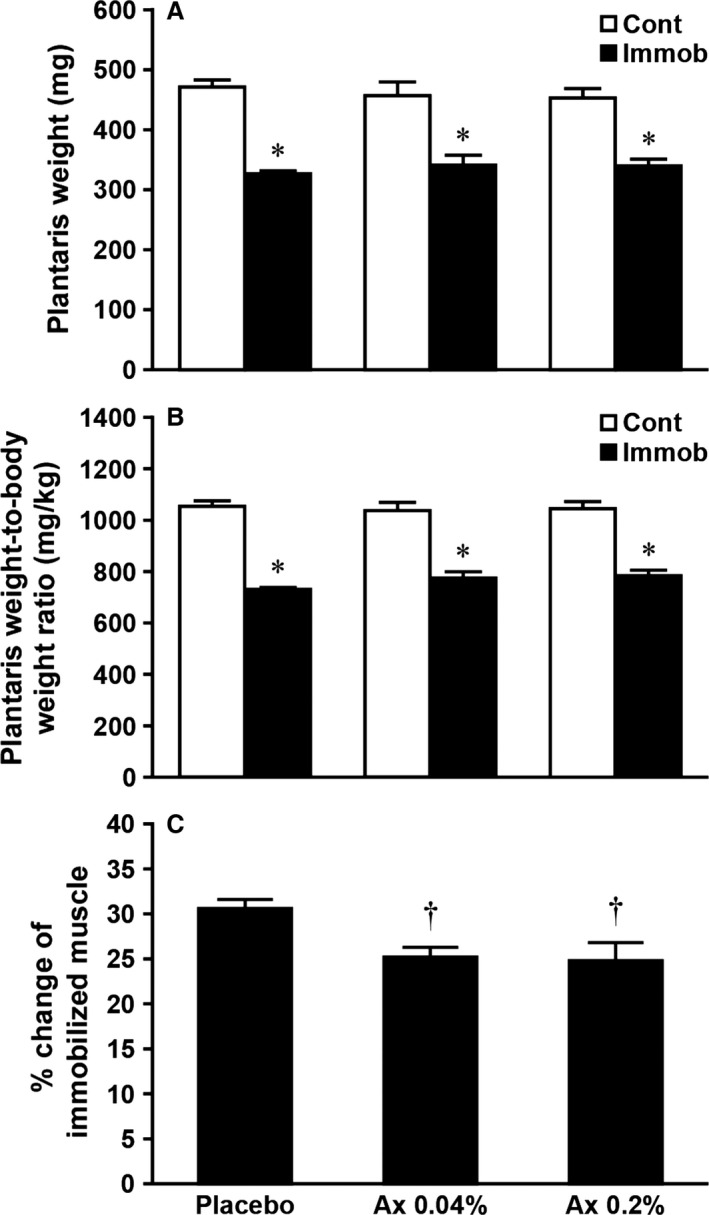
Effects of dietary astaxanthin intake on plantaris muscle weight (A), plantaris weight‐to‐body weight ratio (B), and degree of atrophy induced by hindlimb immobilization in rats (C). Degree of atrophy is calculated by the following formula: Percent weight change from contralateral muscle = (contralateral − immobilized)/contralateral plantaris weight × 100. Values are means ± SE. *n* = 7–8 per group. Cont, contralateral limb; Immob, immobilized limb. See Table 1 for the other abbreviations. *Significantly different between immobilized and contralateral limbs in each group (*P *<* *0.05). ^†^Significantly different from placebo group (*P *<* *0.05).

In all experimental groups, plantaris muscle mass in immobilized limbs was significantly lower compared with those in contralateral limbs (Fig. 1A, *P *<* *0.05). The muscle mass of each leg did not significantly differ among the three groups. Similar results were also noted in plantaris weight‐to‐body weight ratio (Fig. 1B). However, the degree of plantaris muscle atrophy was significantly less in the Ax 0.04% and Ax 0.2% groups than in the placebo group (Fig. 1C, *P* < 0.05).

**Figure 2:**
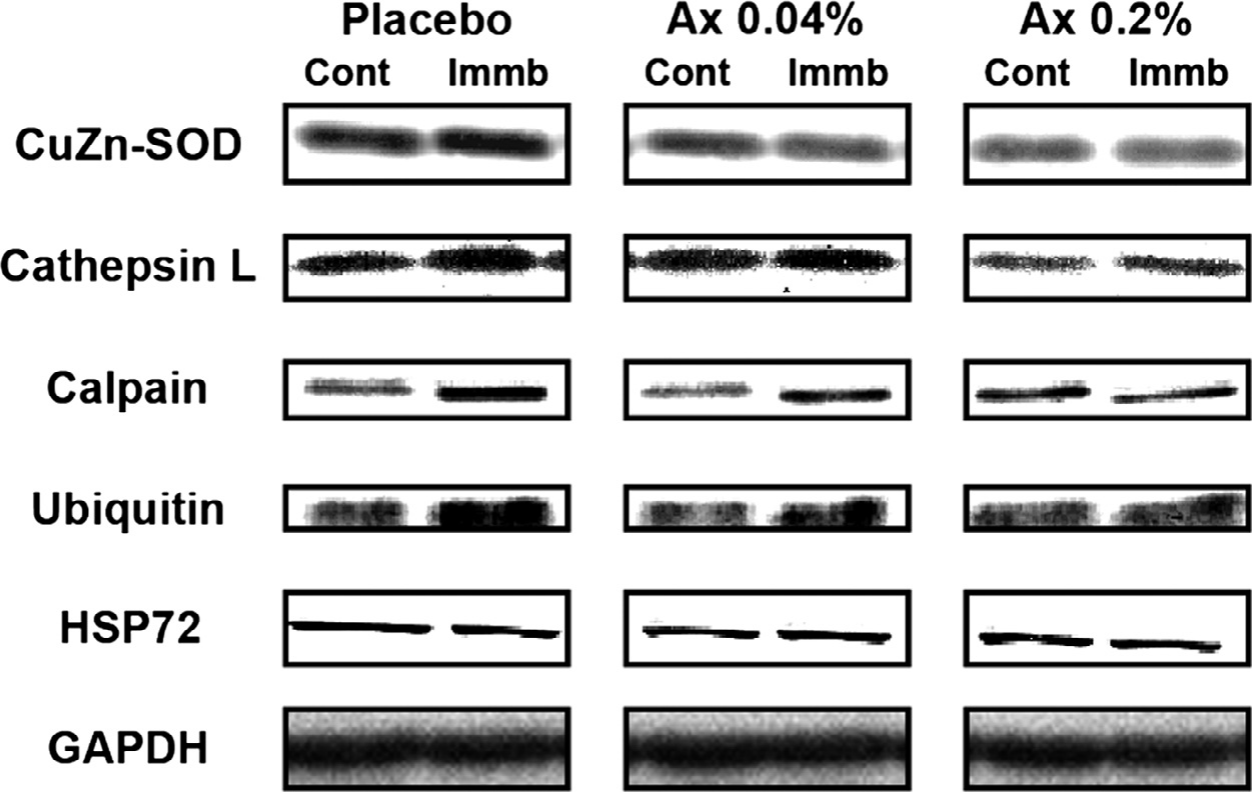
Representative western blot images of CuZn‐superoxide dismutase (CuZn‐SOD), cathepsin L, calpain, ubiquitin, heat‐shock protein 72 (HSP72), and glyceraldehyde‐3‐phosphate dehydrogenase (GAPDH) in atrophied plantaris muscle induced by hindlimb immobilization with or without dietary astaxanthin intake. Definition of abbreviations: Placebo, placebo diet group; Ax 0.04%, 0.04% astaxanthin diet group; Ax 0.2%, 0.2% astaxanthin diet group; Cont, contralateral limb; Immob, immobilized limb.

**Figure 3:**
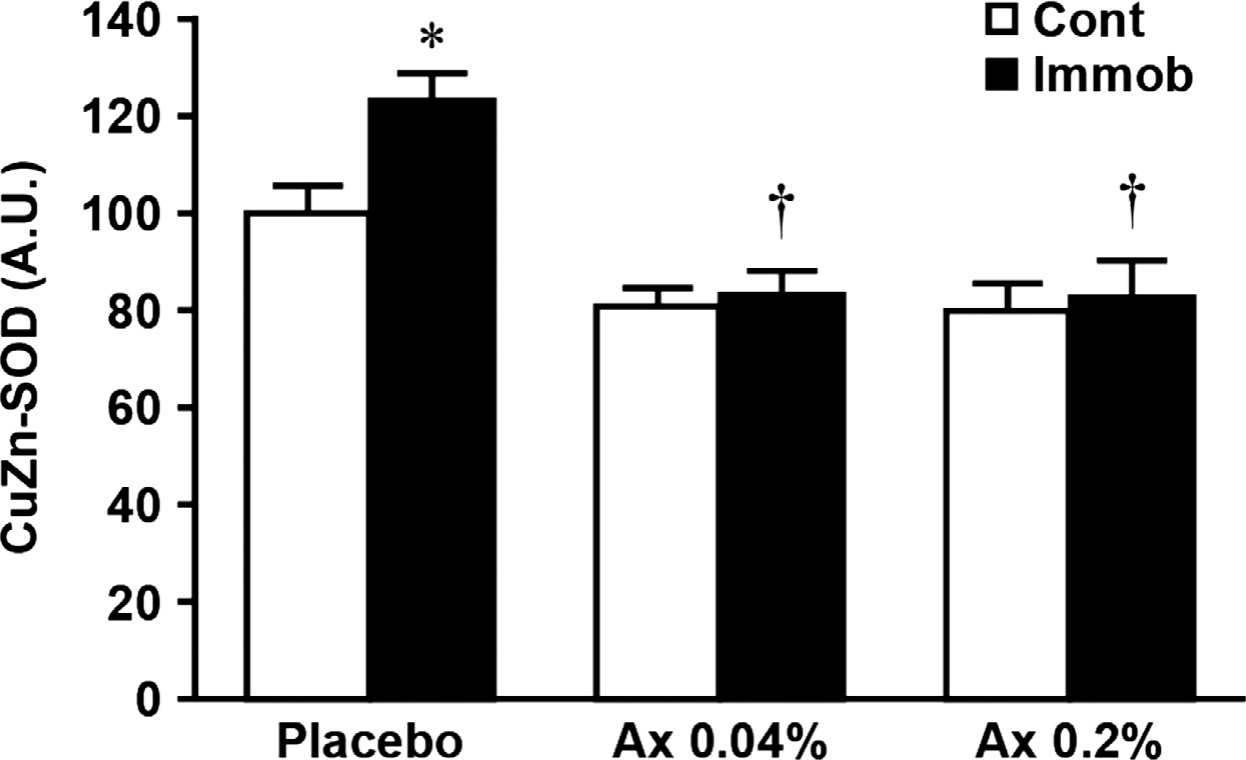
Effects of dietary astaxanthin intake on the expression level of CuZn‐SOD in atrophied plantaris muscle induced by hindlimb immobilization. Values are means ± SE. *n* = 7–8 per group. Arbitrary OD units (A.U.) are expressed as the relative ratio (%) to the nonimmobilized muscles of the placebo diet group. See Table 1 and Figure 1 for the abbreviations. *Significantly different between immobilized and contralateral limbs in each group (*P *<* *0.05). ^†^Significantly different from placebo group (*P *<* *0.05).

The protein level of CuZn‐SOD in the placebo group was significantly higher in atrophied than in control muscles (Figs 2 and 3, *P *<* *0.05), whereas that in the Ax 0.04% and Ax 0.2% groups did not differ between the both muscles. Furthermore, CuZn‐SOD levels in the atrophied muscle were significantly lower in Ax 0.04% and Ax 0.2% than in placebo animals (*P *<* *0.05).

**Figure 4:**
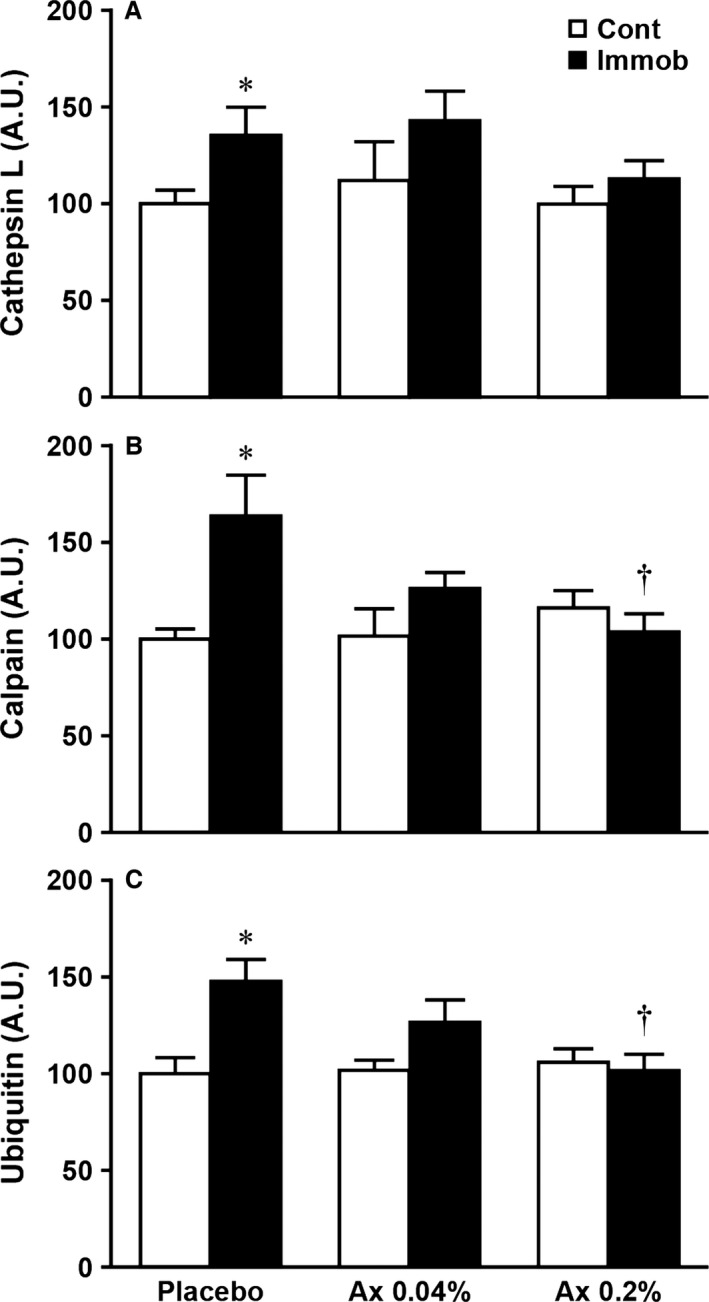
Effects of dietary astaxanthin intake on the protein expressions of cathepsin L (A), calpain (B), and ubiquitin (C) in atrophied plantaris muscle induced by immobilization of rat hindlimb. Arbitrary OD units (A.U.) are expressed as the relative ratio (%) to the nonimmobilized muscles of the placebo diet group. Values are means ± SE. *n* = 7–8 per group. See Table 1 and Figure 1 for the abbreviations. *Significantly different between immobilized and contralateral limbs in each group (*P *<* *0.05). ^†^Significantly different from placebo group (*P *<* *0.05).

**Figure 5:**
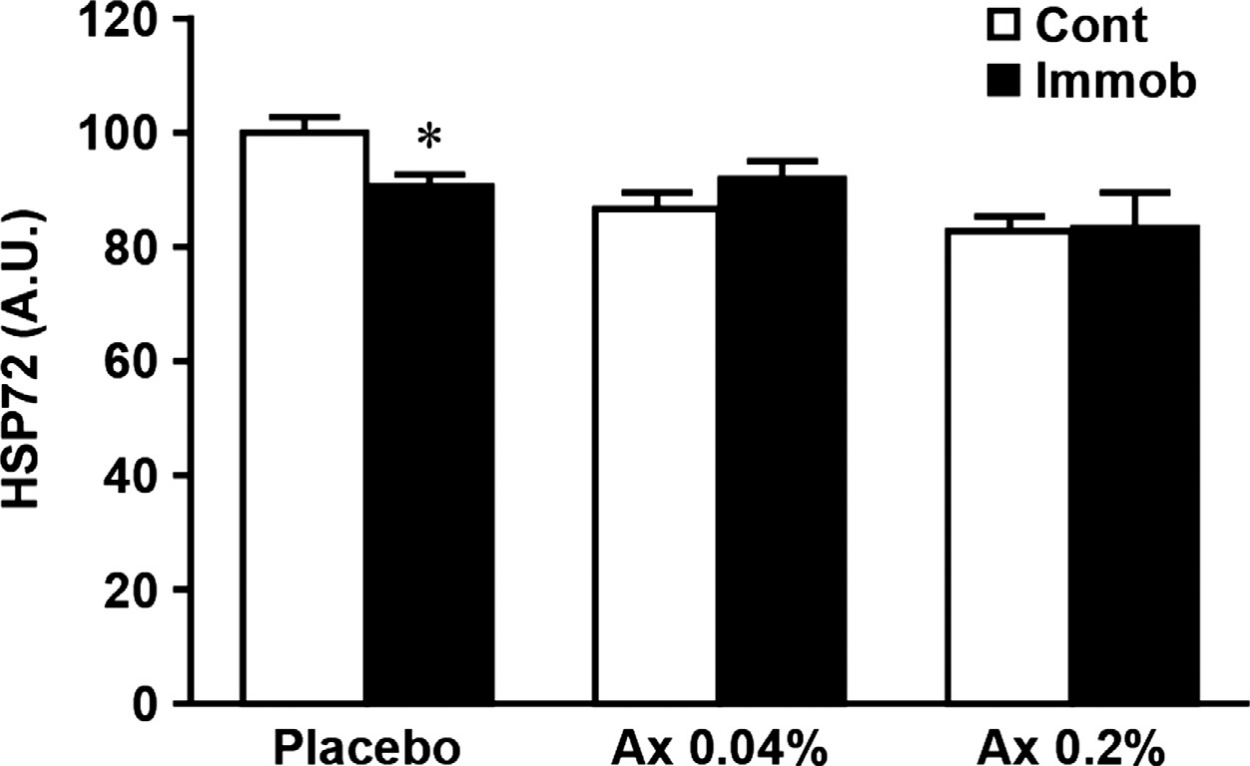
Effects of dietary astaxanthin intake on the expression level of HSP72 in atrophied plantaris muscle induced by hindlimb immobilization. Arbitrary OD units (A.U.) are expressed as the relative ratio (%) to the nonimmobilized muscles of the placebo diet group. Values are means ± SE. *n* = 7–8 per group. See Table 1 and Figure 1 for the abbreviations. *Significantly different between immobilized and contralateral limbs in each group (*P *<* *0.05).

In the placebo group, 10 days of immobilization resulted in a significant increase in the expression of cathepsin L, calpain, and ubiquitin (Figs 2 and 4, *P *<* *0.05) and a significant decrease in the expression of HSP72 (Figs 2 and 5, *P *<* *0.05) in the immobilized muscle, relative to the matched control muscle. However, there were no significant differences in these parameters between control and atrophied muscles in the both astaxanthin‐treated groups. In addition, the expression levels of calpain and ubiquitin were significantly lower in the immobilized muscle of the Ax 0.2% than in the placebo group (Fig. 4B and C, *P *<* *0.05).

## Discussion

### Astaxanthin and muscle atrophy

It is well established that immobilization‐induced oxidative stress such as increased ROS production (e.g., superoxide anion, hydrogen peroxide, and hydroxyl radical) and lipid peroxidation is one of the causes of muscle atrophy (Kondo et al. 1991, 1992, 1993a,b, 1994). Unfortunately, the precise mechanism of this event is still unclear. Nonetheless, it seems that oxidative stress acts as an upstream regulator of the activation of proteolysis via three major proteolytic pathways (lysosomal proteases, calpains, and the ubiquitin–proteasome system) in skeletal muscle during inactivity including immobilization (Ikemoto et al. 2002a; Sugiura et al. 2006; Min et al. 2011; Talbert et al. 2013). In fact, previous studies have demonstrated that the induction of oxidative stress stimulated protein degradation in skeletal muscle myotubes by up‐regulating cathepsins, calpains, and the ubiquitin–proteasome system (Li et al. 2003; Nakashima et al. 2004). Consistent with these concepts, this study showed that immobilization caused plantaris muscle atrophy along with the up‐regulation of CuZn‐SOD, which reflects the level of cytosolic superoxide anions, and proteases (cathepsin L, calpain, and ubiquitin) (Figs 1, 3 and 4).

Astaxanthin is a natural antioxidant and exhibits more effective antioxidant activities than other antioxidants (Kurashige et al. 1990; Miki 1991; Goto et al. 2001). We and others previously demonstrated an inhibitory effect of astaxanthin on oxidative stress and/or oxidative damage in skeletal muscle after intense exercise (Aoi et al. 2003) and during inactivity induced by hindlimb unloading (Kanazashi et al. 2013, 2014; Yoshihara et al. 2016). Although the underlying mechanism of the antioxidant action of astaxanthin on skeletal muscle remains unclear, it appears that astaxanthin can act as scavenger and/or quencher of ROS and free radicals (Kurashige et al. 1990; Miki 1991; Kobayashi 2000; Goto et al. 2001). In this study, we found that astaxanthin supplementation at both concentrations prior to and during immobilization period significantly attenuated the rate of plantaris muscle atrophy (Fig. 1C) and the up‐regulation of CuZn‐SOD (Fig. 3). Moreover, this is the first study showing that a significant increase in cathepsin L, calpain, and ubiquitin levels in response to immobilization was prevented by dietary intake of astaxanthin (Fig. 4). These results are consistent with our recent study using rat hindlimb unloading model (Yoshihara et al. 2016) and the results from administration of other antioxidants during muscular inactivity (Ikemoto et al. 2002a; Sugiura et al. 2006; Servais et al. 2007; Min et al. 2011; Talbert et al. 2013). Therefore, all aforementioned findings suggest that dietary astaxanthin intake reduced immobilization‐induced oxidative stress, at least production of superoxide anion and subsequent production of hydrogen peroxide by CuZn‐SOD, through the ROS quenching and/or scavenging effects of astaxanthin, which then resulted in an attenuation of plantaris muscle atrophy by inhibiting proteolysis via three major proteolytic pathways. In contrast to our findings, Kanazashi et al. reported that astaxanthin supplementation alone did not prevent hindlimb unloading‐induced soleus muscle atrophy in rats (Kanazashi et al. 2013, 2014). Although we have no clear explanation of these divergent results, these discrepancies may be due to differences in atrophy model (i.e., unloading vs. immobilization), fiber‐type differences between soleus and plantaris muscles and/or duration of astaxanthin administration.

It should be noted that dietary intake of astaxanthin did not completely inhibit immobilization‐induced plantaris muscle atrophy (Fig. 1), even though the up‐regulation of proteases was prevented (Fig. 4). In general, skeletal muscle mass is determined by the balance between protein synthesis and degradation. Although we did not measure any parameters involved in protein synthesis in this study, it has been demonstrated that the rate of protein synthesis in skeletal muscle was significantly reduced by hindlimb immobilization in rats (Booth and Seider 1979; Tucker et al. 1981). Therefore, it was speculated that protein balance in plantaris muscle might remain negative during the immobilization period when astaxanthin was administrated daily. Further studies are needed to clarify this issue.

### Astaxanthin and HSP72

HSP72 is one of the stress proteins induced by various stresses such as heat stress and ROS. This protein acts as molecular chaperones, has strong cytoprotective effects, and contributes to preventing protein aggregation and refolding damaged proteins (Powers et al. 2001; Kregel 2002). Several studies reported that the expression level of HSP72 in skeletal muscle was significantly reduced in response to inactivity (Ku et al. 1995; Naito et al. 2000; Selsby et al. 2007). In addition, Ku et al. (1995) reported that down‐regulation of HPS72 may be associated with the reduction of protein synthesis through the slowing of nascent polypeptide elongation during muscle atrophy. Therefore, based on the roles of HPS72 and the results of Ku et al. (1995), it is possible that inactivity‐induced decrease in HSP72 levels may be one of the causes of muscle atrophy.

In agreement with previous reports (Ku et al. 1995; Naito et al. 2000; Selsby et al. 2007), the expression level of HSP72 in the placebo group was significantly decreased by immobilization in our study (Fig. 5). However, the immobilization‐induced decrease of HSP72 levels was not observed in both astaxanthin‐treated groups. Although the mechanisms of this effect are still unclear, these findings suggest that maintenance of HSP72 levels induced by dietary intake of astaxanthin could maintain the beneficial functions of HSP72 described above during immobilization, which resulted in reducing plantaris muscle atrophy.

### Dose‐dependent effects of astaxanthin

Based on the results of Ikemoto et al. (2002a) who reported that only high‐dose administration of cysteine (140 mg per rat) had inhibitory effects on muscle atrophy, TBARS concentrations, and ubiquitination of muscle proteins induced by hindlimb unloading, we also examined the effects of dietary astaxanthin intake at two different concentrations (0.04% or 0.2%) on immobilization‐induced plantaris muscle atrophy. We found that only Ax 0.2% group showed a significant reduction of calpain and ubiquitin levels in atrophied muscle compared with the placebo group (Fig. 4B and C). However, a protective effect of astaxanthin at both concentrations on atrophy of plantaris muscle was similar in this study (Fig. 1C). Therefore, these findings indicate that dietary astaxanthin intake at a low dose (6.5–10.1 mg per day per rat) may be beneficial for attenuating immobilization‐induced plantaris muscle atrophy.

## Conclusions

This study demonstrated that dietary astaxanthin intake attenuated skeletal muscle atrophy due, in part, to reducing oxidative stress, as well as cathepsin L, calpain, and ubiquitin levels, and maintaining HSP72 expressions. In addition, a low‐dose intake of astaxanthin (6.5–10.1 mg per day per rat) was enough to induce these positive effects. Our data strongly suggest that astaxanthin intake may be an effective countermeasure against disuse muscle atrophy.

## Conflict of Interest

None declared.
